# Revisiting the genomes of herpesviruses

**DOI:** 10.7554/eLife.54037

**Published:** 2020-01-16

**Authors:** Bhupesh K Prusty, Adam W Whisnant

**Affiliations:** Institute for Virology and ImmunobiologyJulius-Maximilians-Universität WürzburgWürzburgGermany

**Keywords:** human herpesvirus 6, RNA-seq, ribosome profiling, genome annotations, herpesvirus, transcriptomics, Virus

## Abstract

Combining integrative genomics and systems biology approaches has revealed new and conserved features in the genome of human herpesvirus 6.

**Related research article** Finkel Y, Schmiedel D, Tai-Schmiedel J, Nachshon A, Winkler R, Dobesova M, Schwartz M, Mandelboim O, Stern-Ginossar N. 2020. Comprehensive annotations of human herpesvirus 6A and 6B genomes reveal novel and conserved genomic features. *eLife* 9:e50960. doi: 10.7554/eLife.50960

Herpesviruses cause a range of human diseases but many factors complicate the efforts made to precisely map the size and origin of RNA transcripts coded by these pathogens. For example, some mRNAs can code for more than one protein, coding sequences may overlap with each other, and the genes that are expressed may change depending on cell types or stages in the viral cycle. Moreover, the level of expression can greatly vary from gene to gene, which makes it difficult to distinguish between rare viral transcripts and other genetic products that accumulate in infected cells and during viral replication. In fact, in most herpesviruses, the majority of the genome is transcribed to some degree, yet only the most highly expressed or genomically isolated units are readily detectable.

Several new techniques have allowed researchers to bypass these problems to better annotate the genomes of herpesviruses. A tailored RNA sequencing method called cRNA-Seq, which enriches for the 5’ ends of RNA transcripts, has allowed the mapping of transcription start sites; in parallel, ribosome profiling (Ribo-Seq) has helped to highlight translational start sites. Combined, these approaches have revealed dozens to hundreds of new genes in herpesviruses such as the human cytomegalovirus (Stern-Ginossar et al., 2012) and the Kaposi’s sarcoma-associated herpesvirus (Arias et al., 2014). When paired with long-read sequencing platforms (which provide additional information about the 3’ ends of transcripts), the new methods have also led to a better understanding of a number of pathogens in the herpes family. Now, in eLife, Noam Stern-Ginossar and colleagues at the Weizmann Institute of Science and the Hebrew University Hadassah Medical School – including Yaara Finkel as first author – report new insights into human herpesvirus 6A and 6B (Finkel et al., 2020).

The results help to correct and complement previous textbook genome annotations for herpesviruses. Due to the technical limitations of the time, the exact beginnings of many transcripts and coding sequences were assigned a priori, and inclusion into published gene lists relied on rather conservative criteria. For instance, a sequence was classified as an open reading frame (the part of a genetic sequence that can potentially be translated) if it had more than 100 amino acids and started with an AUG codon. Instead, Finkel et al. demonstrate that roughly one-third of open reading frames in human herpesvirus 6A and 6B contain alternative start codons, which are also used by eukaryotes and other herpesviruses (Kearse and Wilusz, 2017; Arias et al., 2014). For instance, strains of human cytomegalovirus can have different start codons for a given gene, which may influence biological properties (Brondke et al., 2007); such questions can now be investigated in herpesvirus 6A and 6B .

Another exciting finding is the identification of hundreds of short, internal or upstream open reading frames (Figure 1). The proteins encoded by many of these sequences are likely to be too small to have direct functions. However, some of these short open reading frames are close to (or overlap with) longer coding sequences, suggesting that they may regulate translation – particularly during the later stage of viral gene expression, when homeostasis in the host cells is most disrupted. Finkel et al. observed that several of these open reading frames are also transcribed in human cytomegalovirus, indicating important conserved roles across the family of viruses that herpesvirus 6A and 6B belong to.

**Figure 1.**
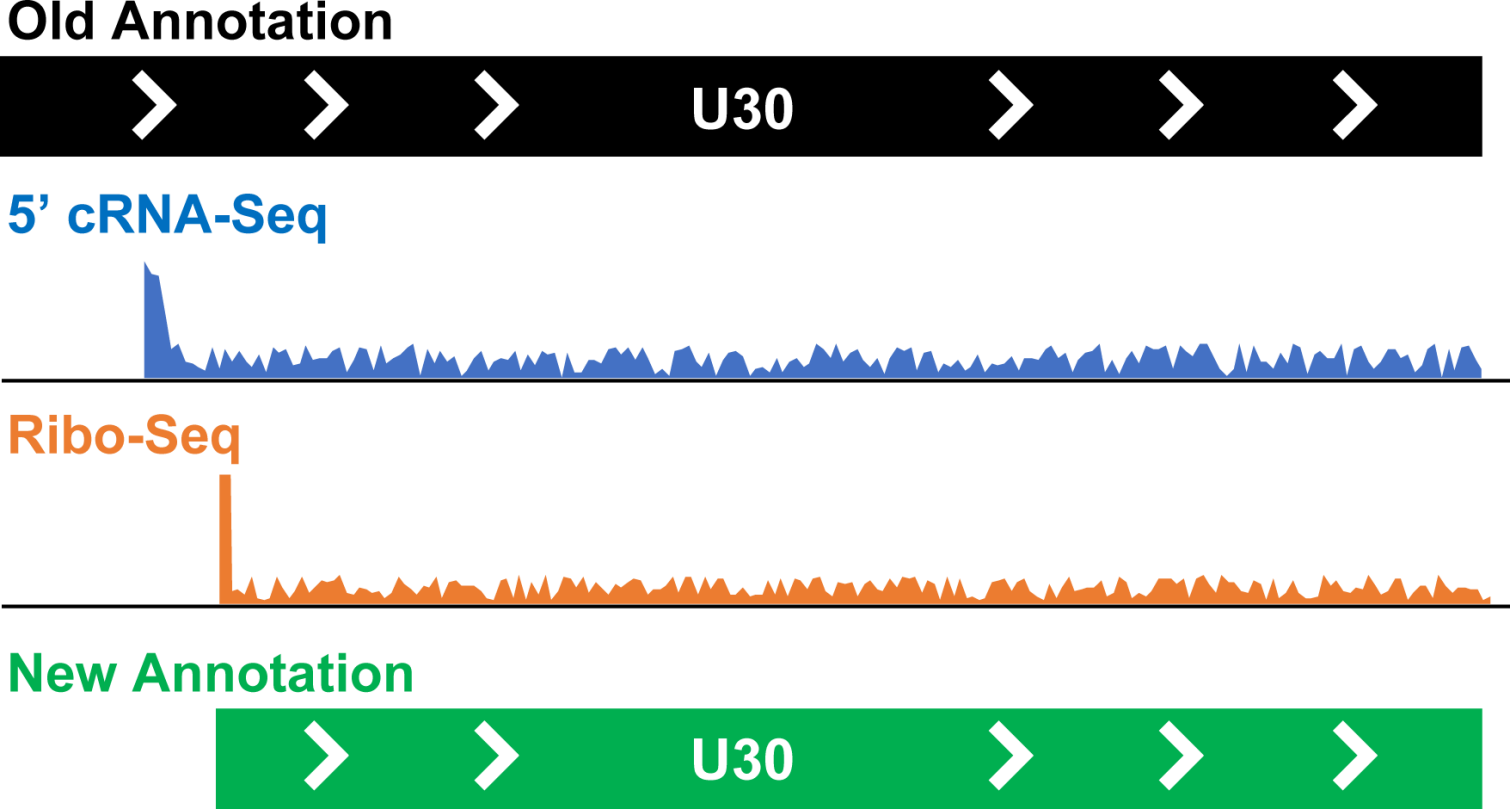
Taking a closer look at the genomes of human herpesviruses 6. Finkel et al. have used a combination of techniques to reannotate the genomes of human herpesviruses 6A and 6B. They have identified new open reading frames (268 in human herpesvirus 6A and 216 in human herpesvirus 6B) and corrected the annotation of existing frames (10 in human herpesvirus 6A and 11 in human herpesvirus 6B). The figure shows how an open reading frame called U30, which codes for an important protein in both human herpesvirus 6A and 6B, was reannotated. Data from Ribo-Seq (orange) revealed that the start of the open reading frame was downstream of what was expected based on the previous annotation (black) or cRNA-Seq information (blue), leading to a new, more accurate annotation for this sequence (green).

Combining several methods that can pinpoint both translational and transcriptional start sites – as Finkel et al. did – is particularly important because modern sequencing protocols are sensitive enough to identify rare transcription events, but they cannot distinguish between ‘real’ transcriptional units and biological artifacts. Whole-genome conclusions based on one technique or method of analysis are heavily influenced by experimental noise, technical limitations and even the specific algorithm used to interpret the data. For instance, estimates of the exact number of transcriptional start sites in human cytomegalovirus vary by thousands between studies that use different methods (Stern-Ginossar et al., 2012; Parida et al., 2019); in herpes simplex virus, these numbers can vary by over six-fold (Tombácz et al., 2019; Depledge et al., 2019).

While our appreciation of the coding capacity of pathogens increases, efforts must be made to integrate newly identified gene products into already established nomenclatures. The first waves of new annotations using high-throughput techniques will probably be revised as sequencing technology and analysis techniques improve, and the results are validated in the lab. In particular, new algorithms that can better distinguish signal-to-noise values could help to identify hundreds of additional peptides in a second revision of the human cytomegalovirus genome (Erhard et al., 2018). As our ability to sequence deeper develops, multifaceted studies such as the one by Finkel et al. will provide an excellent framework to help distinguish between rare functional events and technical noise when re-examining herpesvirus genome annotations.
